# Connecting dots between nucleotide biosynthesis and DNA lesion repair/bypass in cancer

**DOI:** 10.1042/BSR20231382

**Published:** 2024-09-23

**Authors:** Jackson C. Lin, Ayobami Oludare, Hunmin Jung

**Affiliations:** The Division of Medicinal Chemistry, School of Pharmacy, The University of Connecticut, Storrs, Connecticut 06269, U.S.A.

**Keywords:** Cancer, DNA damage response, drug discovery and design, drug resistance, Nucleotide biosynthesis

## Abstract

Purine and pyrimidine nucleotides are crucial building blocks for the survival of cells, and there are layers of pathways to make sure a stable supply of them including *de novo* nucleotide biosynthesis. Fast-growing cells including cancer cells have high demand for nucleotide, and they highly utilize the nucleotide biosynthesis pathways. Due to the nature of the fast-growing cells, they tend to make more errors in replication compared with the normal cells. Naturally, DNA repair and DNA lesion bypass are heavily employed in cancer cells to ensure fidelity and completion of the replication without stalling. There have been a lot of drugs targeting cancer that mimic the chemical structures of the nucleobase, nucleoside, and nucleotides, and the resistance toward those drugs is a serious problem. Herein, we have reviewed some of the representative nucleotide analog anticancer agents such as 5-fluorouracil, specifically their mechanism of action and resistance is discussed. Also, we have chosen several enzymes in nucleotide biosynthesis, DNA repair, and DNA lesion bypass, and we have discussed the known and potential roles of these enzymes in maintaining genomic fidelity and cancer chemotherapy.

## Introduction

Nucleotides, along with nucleosides and nucleobases, are crucial building blocks for cell survival, and they are needed in replication, transcription, and many other cellular processes. The need for nucleotides is naturally higher in fast-growing cells such as cancer cells, and many of the early anticancer agents were not surprisingly nucleotide analogs such as 5-fluorouracil (5-FU), which is still a first line chemotherapeutic agent in several cancers including colorectal cancer (CRC) [1,2]. There are lots of other nucleotide-analog anticancer drugs such as 6-mercaptopurine [3,4], 6-thioguanine [5,6], and gemcitabine [7,8], and we will be discussing these nucleotide analog cancer chemotherapeutic agents along with the crucial enzymes and pathways closely related to them more in detail in this review.

The genomic integrity of human cells is constantly threatened by a wide variety of factors from exogenous and endogenous agents that can cause DNA damage. Some of the well-known reactions by which DNA damage can be formed are alkylation, oxidation, and deamination. Since inosine monophosphate (IMP), whose base is hypoxanthine (HX), is a key intermediate in *de novo* purine nucleotide biosynthesis, which generates adenosine monophosphate (AMP) and guanosine monophosphate (GMP), *de novo* nucleotide biosynthesis is crucial for DNA replication. Not just the biosynthesis of nucleotides, the phosphorylation of nucleotides, from nucleoside to mono and triphosphate, also plays an important role especially for the incorporation of nucleotide. The crucial roles of nucleotide biosynthesis and maintaining the proper level of triphosphate form of nucleotides will be further discussed in this review.

Cells have layers of defense mechanism to protect themselves from these threats, and one of the ways is to remove the triphosphate form of non-canonical nucleotide. The triphosphate form of nucleotides is one of the risk factors for genome integrity, and the cellular level of the triphosphate form of non-canonical nucleotides is tightly regulated. We will be discussing the relationship between *de novo* nucleotide biosynthesis, including the action of regulating the level of triphosphate form of nucleotides, and DNA repair/bypass more in detail later in this review.

### Nucleotide analog anticancer drugs

**Figure 1:**
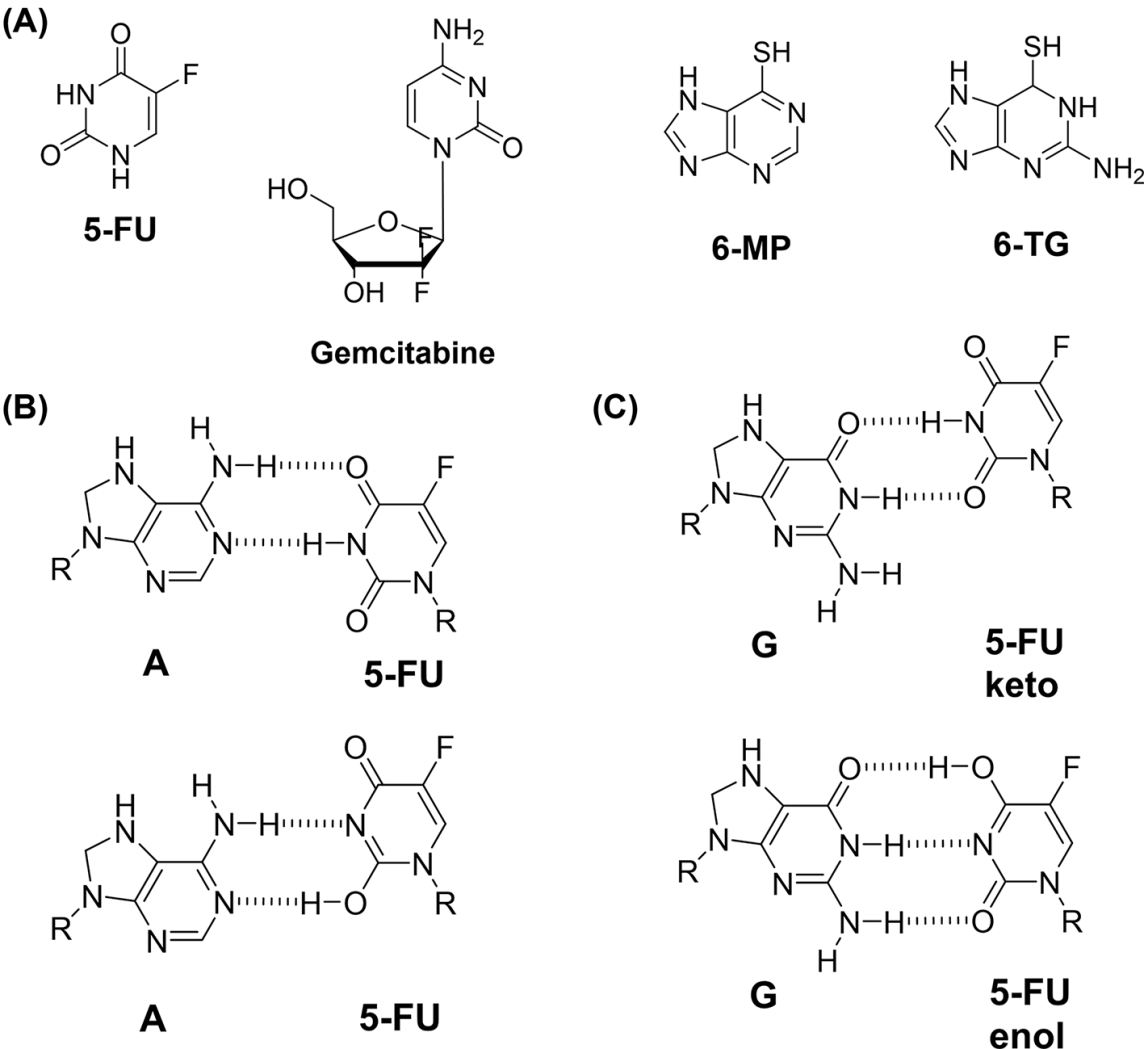
The chemical structures of nucleotide analog drugs and the base pair interactions of 5-FU (**A**) The chemical structures of nucleotide analog anticancer agents, 5-FU, gemcitabine, 6-MP, and 6-TG. (**B**) 5-FU can form a Watson-Crick base pair (top) or wobble base pair (bottom) with adenine. (**C**) 5-FU can also form a wobble base pair via a keto tautomer of 5-FU (top) and a Watson-Crick base pair via an enol tautomer of 5-FU (bottom).

Nucleotide (nucleoside/nucleobase included) analogs have long been used ever since the development of the analog of uracil, 5-fluorouracil (5-FU). Nucleotides are a crucial building block for the synthesis of DNA and RNA, and they are indispensable for the survival of cells. That is why there have been a lot of drugs whose chemical structures are similar to nucleotides, and some of the FDA-approved nucleotide analogs that have been used for treating cancer for years have been compiled in Table 1. Among those nucleotide analogs, we will be discussing some of the milestone drugs, 5-FU, gemcitabine, 6-mercaptopurine (6-MP), and 6-thioguanine (6-TG), that are being actively used but have serious shortcomings that need to be overcome such as drug resistance. The chemical structures of some of these drugs are shown in Figure 1A, and they are analogs of uracil (5-FU), cytidine (gemcitabine), adenine (6-MP), or guanine (6-TG).

**Table 1:** List of selected nucleobase/nucleoside analog drugs approved by FDA

Name	Modification	Target cancer	FDA approval status
5-Fluorouracil (5-FU)	Fluorination on uracil	Colon, esophageal, stomach, pancreatic, breast, and cervical cancers	FDA approved (1962)
Floxuridine	Fluorination on cytosine	Colon cancer	FDA approved (1970)
Cytarabine	Replacement of the ribose ring with arabinose	Acute myelogenous leukemia, Lymphoblastic leukemia	FDA approved (1969)
Gemcitabine	Fluorination on the ribose ring of cytosine	Pancreatic, lung, breast, and bladder cancers	FDA approved (1996)
Azacitidine	Azotation on cytosine	Myelodysplastic syndromes, Acute myeloid leukemia	FDA approved (2004)
Decitabine	Azotation on cytosine, Deoxyribose ring	Myelodysplastic syndromes, Acute myeloid leukemia	FDA approved (2006)
Cladribine	Chlorination on adenine	Hairy-cell leukemia, Non-Hodgkin lymphoma	FDA approved (1993)
6-Mercaptopurine (6-MP)	Thiol addition on adenine	Acute lymphoblastic leukemia	FDA approved (1953)
6-Thioguanine (6-TG)	Thiol addition on guanine	Acute myeloid leukemia, B-cell acute lymphoblastic leukemia	FDA approved (1966)
Fludarabine	Fluorination on adenine, Replacement of the ribose ring with arabinose	Chronic lymphocytic leukemia	FDA approved (1991)
Nelarabine	Methylation on guanine, Replacement of the ribose ring with arabinose	T-cell acute lymphoblastic leukemia and lymphoma	FDA approved (2005)

#### 5-Fluorouracil (5-FU)

Ever since Heidelberger et al. developed an analog of uracil, 5-fluorouracil (5-FU), in the late 1950s [9], 5-FU has been widely used for various cancers [1,2,10]. In cells, including cancer cells, 5-FU is converted by the actions of the enzymes, thymidine phosphorylase (TP) and thymidine kinase (TK) into 5-fluoro-2′-deoxyuridine-5′-mono-phosphate (FdUMP), to inhibit an enzyme called thymidylate synthase (TS) [11]. FdUMP can be a pseudo-substrate for TS and form a dead-end complex with the cysteine residue of TS and another substrate of TS, 5,10-methylenetetra-hydrofolate (MTHF). FdUMP, along with FUMP, can also be further processed to FdUTP or FUTP to exert cytotoxicity via direct incorporation into DNA and RNA [12]. Recently we showed that 5-FU can form either a Watson-Crick base pair or wobble base pair with dA (Figure 1B) and dG (Figure 1C) in the active site of polη. Other than the general mechanisms of drug resistance such as multidrug efflux transporters, there are several mechanisms for CRC cells to acquire resistance to 5-FU. One of the potential mechanisms is the removal of 5-FU by several DNA glycosylases, and the 5-FU removal by uracil DNA glycosylase (UDG) in cancer cells was also reported to be related to 5-FU resistance [13,14], and we will also be discussing one of the DNA repair processes, base excision repair (BER).

Another mechanism for 5-FU resistance is TS overexpression in response to 5-FU [15–17]. TS is one of the three enzymes comprising the thymidylate cycle along with dihydrofolate reductase (DHFR) and SHMT, and there are two isoforms of SHMT, cytosolic SHMT1 and mitochondrial SHMT2. All three enzymes are closely connected, and DHFR inhibitor, methotrexate (MTX) for example, was reported to bring a significantly better clinical outcome for 5-FU compared with 5-FU alone [12]. Also, FdUMP forms a complex with the product of SHMT reaction, MTHF, and the inhibition of SHMT can lower the availability of MTHF significantly and increase the sensitivity for 5-FU. Furthermore, SHMT2 has been reported to be up-regulated and overexpressed in the cancer cells and a potential cancer driver gene [18–20], and low expression of SHMT2 was shown to be closely related to 5-FU resistance in CRC [21]. SHMT2 was also reported to play a central role in serine metabolism in mitochondria, which leads to 5-FU resistance by boosting nucleotide biosynthesis [22]. Targeting both SHMT1 and SHMT2 together was recently shown to be more effective in T-cell acute lymphoblastic leukemia (T-ALL) therapy [23], and the inhibitors working on both SHMT1 and 2 were sensitive on methotrexate-resistant cell lines as well [23]. We will be discussing SHMT further in the later section, and based on the prior research, UDG along with SHMT, especially SHMT2, can be a great strategy for developing a novel 5-FU combination chemotherapy.

#### Gemcitabine

Gemcitabine is a deoxycytidine analog commonly used as a first- or second-line chemotherapeutic agent for several cancers including pancreatic cancer [24], non-small cell lung cancer [25], ovarian cancer [26], and breast cancer [27]. Like many other nucleotide base analogs, it mainly functions by being incorporated into DNA and arresting the cell cycle in the S phase [28]. Specifically, it competes against deoxycytidine triphosphate (dCTP). Once incorporated into DNA, it causes DNA polymerase to halt, and the inhibition of DNA elongation will eventually lead to cell death. Gemcitabine is also a potent inhibitor of ribonucleotide reductase (RR) and because of depleted dCTP, deoxycytidylate deaminase (DCTD) is inhibited as well. RR is responsible for converting CTP into dCTP and its inhibition, which lowers dCTP pools, increases the chance of gemcitabine being incorporated into DNA [29,30].

Activation of gemcitabine involves being phosphorylated to gemcitabine monophosphate (dFdCMP) by deoxycytidine kinase (DCK). It will then be further phosphorylated into gemcitabine diphosphate (dFdCDP) by pyrimidine nucleoside monophosphate kinase (NMPK), then into gemcitabine triphosphate (dFdCTP) by nucleoside diphosphate kinase [28,29,31,32]. One issue with gemcitabine is that cancers appear to become resistant to gemcitabine quite easily after exposure. One known mechanism of resistance is that the cells can become deficient in DCK, which is the limiting step when it comes to gemcitabine activation. Another mechanism of resistance is the down-regulation of nucleoside transporters on the cancer cells. Due to gemcitabine being hydrophobic, it cannot cross the cell membrane without the assistance of these transporters. The primary transporter of gemcitabine is hENT1 (SLC29A1) but can also be transported by hENT2 (SLC29A2), hCNT1 (SLC28A1), and hCNT3 (SLC28A3). Cells can also up-regulate cytidine deaminase (CDA) or DCTD, which is responsible for deaminating gemcitabine to 2,2-difluorodeoxyuridine (dFdU) [30–32]. Up-regulation of RR, which produces dNTPs, and thymidylate synthase (TS), which is responsible for the early stages of DNA biosynthesis, helps increase dCTP pools to compete with gemcitabine and decrease misincorporation [29].

Watchers et al. has excluded the possibility of nonhomologous end-joining as a mechanism of the repair of DNA double-strand break (DSB) caused by gemcitabine but determined that gemcitabine may actually affect homologous recombination (HR)-mediated DSB repair and base excision repair (BER) [33]. Since BER relies on dNTP levels, the possibility of gemcitabine inhibiting BER is supported by evidence of gemcitabine inhibiting RR and DCTD [33]. It was also shown that the sensitivity of some cancer cell lines to gemcitabine and another cytidine analog, cytarabine, was increased by the deficiency of *MBD4* gene, which encodes methyl-CpG binding domain 4 (MBD4) [34]. On the other hand, when XRCC1 (X-ray repair cross-complementing) mutant cells were treated by gemcitabine, they still showed radio-sensitization. XRCC1 plays an important role in BER, so this shows that a fully functional BER process is not required to induce radio-sensitization [33]. Gemcitabine has been found to inhibit nucleotide excision repair (NER) as well by inhibiting Gadd45, which mediates NER through DNA methylation and epigenetic activation. Once gemcitabine is incorporated into DNA, it was reported to undergo masked chain termination, which prevents exonucleases from excising gemcitabine [35,36].

Rad51 homologs, XRCC2 and XRCC3, play a role in HR, and Rad51 mutants did not show increased radiosensitivity when treated with gemcitabine. This means that HR is likely a target of gemcitabine in normal cells, which is what induces radiosensitivity [33]. Gemcitabine has also been shown to down-regulate Rad51 and accumulate unrepaired DNA damage and down-regulate MSH2 (involved in DNA damage binding and mismatch repair), EXO1 (involved in mismatch repair), and PRKDC, which is involved in double-strand break repair [37].

DNA translesion synthesis (TLS) polymerase activity has shown resistance to certain anticancer drugs such as platinum-based therapies. There has been evidence that DNA Polymerase η (Polη) deficiency has resulted in increased sensitivity to gemcitabine and cisplatin. Polη can resume halted DNA replication that can be caused by drugs like gemcitabine. There is some correlation between Polη expression and resistance to cisplatin and gemcitabine, indicating that Polη levels could be a predictor for resistance to platinum of gemcitabine-based therapy [38,39].

#### 6-Thiopurine (6-mercaptopurine & 6-thioguanine)

6-Mecarptopurine (6-MP) and 6-thioguanine (6-TG) are well-studied chemotherapeutic agents frequently used since the 1950s to treat cancer in patients (Table 1, [40]). 6-MP and 6-TG are thiopurine prodrugs and purine nucleobase analogs requiring extensive metabolism before initiating their anti-proliferative and antineoplastic effects which work by being incorporated into the DNA, disrupting the biochemical process of endogenous purines, DNA damage, and ultimately apoptosis [41,42]. They have been identified as important drugs for the treatment of childhood acute lymphoblast leukemia (ALL) [40] and as immunosuppressants after organ transplants. 6-MP is known for its cytotoxicity in childhood acute lymphoblastic lymphoma by way of the incorporation of its metabolite into the DNA and its mechanism of inhibition of the *de novo* purine biosynthesis pathway [43,44]. Being a prodrug, it has no physiological activity, however, upon administration, it undergoes biotransformation through intestinal and hepatic metabolisms.

6-MP is well known for its cytotoxicity in childhood acute lymphoblastic lymphoma by way of the incorporation of its metabolite into the DNA and its mechanism of inhibition of the *de novo* purine biosynthesis pathway [43,44]. Being a prodrug, it has no physiological activity, however, upon administration, it undergoes biotransformation through intestinal and hepatic metabolisms. Intracellularly, 6-MP is activated by catalysis through an enzyme called hypoxanthine phosphoribosyl transferase (HPRT)- a widely distributed body enzyme to 6-thioinosine-5′-monophosphate (6-TIMP) which is further hydrolyzed to 6-thioxanthone and 6-TG by inosine monophosphate dehydrogenase (IMPDH) and guanosine monophosphate synthetase (GMPS) respectively. These processes are threatened by methylation by the enzyme thiopurine methyltransferase (TPMT), resulting in the formation of its inactive metabolite methyl mercaptopurine (6-TGNs) [45].

The mechanisms of action of 6-MP are categorized into two major categories. The first is the cytotoxic effect through the fraudulent insertion of its active metabolite into the nucleic acids through a process called ‘base analog incorporation’ [46]. 6-MP, a purine analog possesses a highly similar structure to that of natural purine bases (A, G) that are components of the DNA. During the process of DNA base pairing, 6-MP (due to its similar structure to purines) can be incorporated by DNA polymerase into the growing strand in place of natural purines, resulting in DNA containing 6-MP at specific sites of the DNA. This fraudulent insertion of 6-MP into the DNA causes an accumulation of mutated strands due to a mismatch repair system resulting in DNA strand breakage, apoptosis induction, and ultimately, cytotoxic [47]. The second mechanism of action of 6-MP is the inhibition of the *de novo* purine biosynthesis. Cells especially cancerous cells require a high-energy source to replicate exponentially and utilize ATP for this purpose [48]. ATP, the energy currency of cells is generated as a product of the purine *de novo* biosynthesis (AMP to ATP) and is used to drive processes in cells including cell division [49]. Cancer cells, including leukemia, require high ATP for their exponential cell division process and 6-MP nips this process in the bud at an early stage. This inhibition process is carried out by thioinosinic acid (6-TIMP), the byproduct of the metabolism of 6-MP. 6-TIMP inhibits PRPP amidotransferase, a rate-limiting/ first enzyme in the *de novo* synthesis pathway through a retroinhibition mechanism [50]. This combined with the inhibition of HGPRT, inosine dehydrogenase, and adenyl succinate synthetase which catalyzes the conversion of inosine to 5′adenylic acid (AMP) significantly disrupts the *de novo* biosynthesis process, resulting in deficient levels of AMP and GMP and consequently, the cell division due to the lack of energy (ATP). Also, by inhibiting ATP synthesis, 6-MP stimulates the shutdown of glucose metabolism, leading to energetic stress.

6-thioguanine (6TG), like 6-MP, is another purine analog (guanine analog) cornerstone of ALL treatment with its 6′-hydroxyl group replaced with a sulfhydryl group and it is known for its specific cytotoxicity effects. 6TG is activated through phosphorylation by hypoxanthine-guanine phosphoribosyl transferase (HGPRT) to produce 6-thioguanosine 5′monophosphate (TGMP) [51]. TGMP undergoes a series of extensive metabolisms and is finally converted to deoxy-6-thioguanosine 5′triphosphate (dGS) [52]. Like 6-MP, 6-TG metabolites work in two distinct ways, firstly, aggregation of TGMP disrupts guanine nucleotide synthesis by inhibiting inosine monophosphate dehydrogenase and several enzymes used in purine biosynthesis pathway reducing the number of guanines available for DNA synthesis. The second pathway is the fraudulent incorporation of the triphosphate metabolite nucleotide, deoxy-6-thioguanosine-5′-triphosphate, into DNA or RNA. The sham insertion of the triphosphate nucleotide disrupts cell division during the S-phase, ultimately causing apoptosis.

Despite aggressive chemotherapy regimens for the treatment of ALL, the success rate is approximately 80% in all treated patients. One reason is the therapeutic index of these drugs which like many chemotherapeutic agents is relatively narrow. Another reason is due to multiple mutations that cause resistance to the treatment with purine analogs like 6-MP and 6-TG. Studies have shown that leukemia cells have mutated in ways to fight against drugs like 6-TG and 6-MP by different means of polymorphisms and gene down-regulations and up-regulations [53]. Firstly, TPMT is highly up-regulated in ALL cells which deactivate these purine analogs at high rates through methylation. Secondly, polymorphism of AK3L1, which encodes adenylate kinase and is involved in the formation of AMP, and AK3L1 is severely down-regulated. This down-regulation is also seen in genes like NT5C2 which are crucial in drug actions [54]. Finally, one of the enzymes that catalyze 6-MP to its TGN nucleotide, guanosine monophosphate synthase (GMPS) was down-regulated about 2 folds, thus reducing the production of the toxic metabolites required to elicit its cytotoxicity actions [55].

#### New-generation nucleotide analog anticancer drugs

The problem with the old-generations nucleotide analog drugs is the complications that arise due to the toxicity as well as the advent of resistance of these drugs in the patients. As a result, research is ongoing to improve the shortcomings associated with the older generations of nucleotide analogs [56,57]. The innovation of the new generations aims to target specific cancer cells and tumor environments while reducing the side effects observed with old-generation nucleotide analogs [57]. Here are a few examples of these drugs.

- Clofarabine: Clofarabine is a second-generation nucleoside purine analog drug containing fluorine approved for relapsed or refractory acute lymphoblastic leukemia (ALL) in 2004. Clofarabine combines the favorable activities of previous generation nucleotide analog drugs with distinct improvements including selective toxic effects by preferentially targeting cancer cells over healthy cells, manageable side effects in patients, and reduced resistance emergence [58]. Clofarabine contains fluorine at the 2′-arabino position of Cladribine (its nucleotide analog), and this structural modification increases its stability in highly acidic environments such as in the stomach and plasma environment. Clofarabine exhibits its mechanism of action in two distinct ways. The first is primarily DNA synthesis inhibition where it actively (and potently) competes against dATP for binding to DNA polymerase alpha (polα) and epsilon (polɛ) at the phosphodiester linkages [59], and this results in the disruption of DNA elongation and repair, causing chain termination and replication halt. The second mechanism of action is the inhibition of ribonucleotide reductase activity via an allosteric binding on the regulatory subunit, and this consequently reduces the pool of dCTP, dATP, and dGTP, depleting the cells’ energy levels and halting DNA synthesis [60]. Studies show that clofarabine, when introduced as a pro-drug, significantly reduces the emergence of resistance compared with the old-generation nucleotide analog drugs [61].
- 5-AZA-2-deoxycytidine (decitabine): Decitabine is a DNA methyltransferase inhibitor approved in May 2006 for the treatment of myelodysplastic syndromes (MDS) and is sometimes used in the treatment of acute myeloid leukemia (AML) [62,63]. This pyrimidine analog exhibits its antineoplastic activities of potently inhibiting DNA methylation after being converted intracellularly to a triphosphate form which gets incorporated into the DNA [64]. This DNA incorporation results in the inhibition of DNA methyltransferase (DNMT) through a covalent binding to the enzyme, leading to hypomethylation [65]. Hypomethylation in turn reactivates silenced tumor suppressor genes and induces DNA damage and ultimately apoptosis [66]. Decitabine’s epigenetic mechanism allows it to efficiently and selectively target cancer cells because cancer cells have abnormal DNA methylation patterns. There is an ongoing clinical trial for guadecitabine (SGI-110), which is an investigating new drug and modified analog of decitabine [67,68].
- Nelarabine: This arabinofuranosylguanine prodrug was approved for treating T-cell acute lymphoblastic leukemia in 2005 [69]. This guanine analog gets phosphorylated intracellularly to 9-β-D-arabinofuranosylguanine (Ara-G) which is a highly like endogenous base guanosine but contains modifications that make it resistant to degradation by the enzyme adenosine deaminase. Intracellularly, nelarabine is converted to its active triphosphate form Ara-GTP preferentially in T-lymphocytes. Ara-GTP then gets incorporated into the DNA during DNA synthesis, replacing the guanine nucleotide [70]. This replacement inhibits DNA elongation, resulting in DNA chain termination. It also disrupts RNA function by being fraudulently inserted into the RNA by the RNA polymerases. Since Ara-G lacks the 2′ hydroxyl group present in ribose, it disrupts the RNA strand which alters protein synthesis. The disruption to both DNA and RNA of cancer cells ultimately leads to apoptosis Nelarabine like the other new-generation nucleotide analogs has selective toxicity to only T-lymphocytes (where the cancer is) where its phosphorylation is predominantly occurring, reducing toxicities to other healthy cells. Its chemical composition also significantly improves its efficacy by prolonging the intracellular retention of its active metabolite [71].
- Peptide nucleic acids (PNAs): Over the past three decades, there have been astronomical strides made in the pharmaceutics aspect of the discovery of new-generation nucleotide analog drugs used in cancer treatment to improve their cell targeting and delivery properties. Like nucleotide analogs, PNAs are DNA/RNA analogs in which the sugar-phosphate backbone is replaced by an N-2-aminoethylglycine repeating unit connected via peptide bonds [72]. Using a carboxymethyl spacer, these polyamide chains are linked to the nucleobases via a covalent bond, causing them to pair with the complementary nucleic acid sequence. Interestingly, PNAs lack charged phosphate groups, decreasing the chances of electrostatic repulsion, which strengthens their binding to DNA (about 100 times stronger than the canonical DNA complexes) [73]. The advent of antibody–drug conjugates, and aptamer–drug conjugates, opened an array of possibilities for cancer cell-specific targeting, with A10-3.2-Ara-C conjugate as an example. A10-3.2 is a DNA aptamer that binds to specifically to prostate cancer cell-specific membrane antigen [74]. Many of these drugs are converted to prodrugs as a means to ensure that the drugs get to the right cells and only then is it converted to its active metabolite before eliciting its action [75]. The development of PNAs has been a tremendous breakthrough in cancer therapy. Their complicated structure makes their affinity for their targets highly specific, so much so that a tiny mismatch decreases the PNA binding affinity to its target. Another significant property of PNA is its stability. PNAs exhibit high stability to both thermal and enzymatic activities, and they are resistant to enzymatic degradation and can survive under harsh thermal conditions that are damaging to other drugs [76]. PNAs are also used as a delivery vehicle for gene therapy due to their high affinity and specific binding to the DNA. In such cases, PNAs are used as adapters, aptamers, and other specific targeting molecules to efficiently reach their target [77]. PNAs have also been explored as a gene editing tool where they serve as a guide for CRISPR in a PNA-CRISPR system, and this increases the specificity and binding affinity to the DNA, reducing off-target binding [78]. They can also be used as a gene correction tool where after being coupled with DNA repair mechanism tools/ enzymes, they are designed to specifically target mutations in the DNA and facilitate gene correction [79]. These studies showed that DNA repair mechanisms including nucleotide excision repair (NER) can be employed to edit the gene and this process has leverage over the well-established CRISPR/CAS9 method. PNAs can recruit DNA repair enzymes to the error site and instigate repair mechanisms. Another use of PNAs is as antisense technology. PNAs can be used as antisense oligonucleotides where they bind to complementary mRNA, inhibit translation, in the same vein, effectively silence the gene. PNA-based antisense oligonucleotides can effectively and over a long term inhibit miRNAs which are non-coding RNAs that play a role in gene regulation, hence, inhibiting miRNA functions compared with DNA-based ASOs with significantly lower cytotoxicity in treated cells [80].

### Nucleotide biosynthesis pathways

#### Serine hydroxymethyltransferase (SHMT)

**Figure 2:**
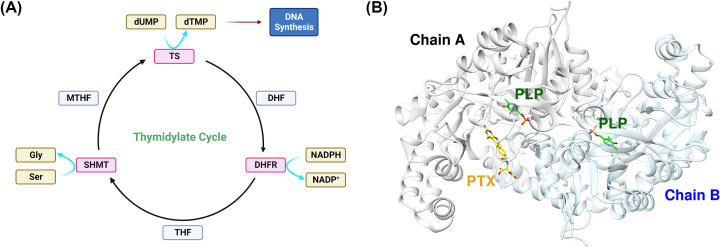
The thymidylate cycle and the enzymes involved in the cycle (**A**) The overview of the thymidylate cycle consisted of serine hydroxymethyltransferase (SHMT), thymidylate synthase (TS), and dihydrofolate reductase (DHFR). (**B**) The crystal structure of SHMT2 homodimer complexed with a cofactor, PLP, and an inhibitor, PTX.

Serine hydroxymethyltransferase (SHMT) is one of the three enzymes in the thymidylate cycle along with dihydrofolate reductase (DHFR) and thymidylate synthase (TS) (Figure 2A). SHMT is an essential enzyme in one-carbon metabolism and in many connected pathways [81,82], but there have been no approved drugs targeting SHMT so far unlike its neighboring enzymes in the thymidylate cycle, TS and DHFR. SHMT has two distinct isoforms, cytosolic SHMT1 and mitochondrial SHMT2, and a lot of studies pointed out that SHMT2 is a highly relevant cancer drug target. For example, SHMT2 inhibition reduced cell proliferation and tumorigenicity in liver cancer [19], and the expression of SHMT2 was up-regulated in colorectal cancer cells [83]. Recent studies have shown that SHMT2 is a key enzyme for nucleotide biosynthesis [84], and the sensitivity of antifolates is closely related to the expression or inhibition of other pathways including base excision repair [85,86]. SHMT2 is reported to form homodimer (Figure 2B), and a cofactor, PLP, and a ligand is bound in the active site of SHMT.

SHMT is an important enzyme in *de novo* nucleotide synthesis as a part of the one-carbon metabolism pathway as well. In addition to nucleotides, these pathways also provide lipids and proteins necessary for cell growth. Within this pathway, SHMT transfers the β-carbon from serine to tetrahydrofolate (THF) to form glycine and 5,10-methylene-THF. 5,10-methylene THF can also be used in the synthesis of methionine or purines [87,88]. Then, 5,10-methylene-THF is used as a methyl donor to create dTMP from dUMP with the assistance of thymidylate synthase (TS) [89]. In this pathway, SHMT is the rate-limiting step in the production of dTMP synthesis, and maintaining a stable rate of SHMT activity can be important to prevent the misincorporation of uracil into the DNA [84]. Something that has been of interest in cancer studies is that SHMT2, but not SHMT1, has been highly expressed in a variety of cancers, and the inhibition of SHMT2 has also been shown to suppress tumor growth and malignancy [89,90]. Many studies have been performed to analyze the other functions of SHMT2 outside of thymidylate synthesis. Like SHMT1, SHMT2 is indispensable in preventing misincorporation of uracil, but in mitochondrial DNA (mtDNA) instead. Cells lacking SHMT2 activity have 67% higher uracil content in mtDNA compared with wild-type cells [89]. SHMT2 plays a role in regulating mitochondrial transfer RNA (tRNA) and formylmethionyl-tRNA pools, and the inhibition of SHMT2 leads to decreased protein synthesis, leading to growth retardation and age-related respiratory defects [89]. SHMT2 and one-carbon metabolism also plays an important role in generating S-adenosylmethionine (SAM), involved in maintaining epigenetic status in the cell. SAM can regulate DNA by promoting DNA methylation, so by inhibiting SHMT2, SAM-related DNA methylation is also inhibited [89,91]. One beneficial function of SHMT2 in cancer cells is that it can help the cancer cells in hypoxic environments. SHMT2 expression is increased under hypoxic conditions in human neuroblastoma cells SHMT2 under hypoxic conditions increases the generation of NADPH and glutathione (GSH), and GSH maintains the cell’s redox status removing reactive oxygen species (ROS) [88,89].

The structure of SHMT1 is a tetramer while SHMT2 is a dimer that transitions into a tetramer when interacting with pyridoxal-5-phosphate (PLP). PLP is the active form of vitamin B6, and its binding is necessary for the function of SHMT [89,90]. SHMT activity can also be dependent on interactions with sirtuin family proteins. SIRT5 can succinylate SHMT at Lys280 to increase enzyme activity [89]. SHMT activity can also be increased when deacetylated at lysine 95 by SIRT2. These two proteins and other proteins of the sirtuin family have been of interest as possible targets in cancer treatment [88,89]. SHMT2 is a direct transcriptional target of the Myc oncogene, and SHMT2 is currently reported as an oncogene and is correlated with tumor progression and poor prognosis in several different cancers [88–90]. Increased SHMT2 is also associated with cancer drug resistance in a few drugs. Interestingly, SHMT2 expression is decreased in the case of 5-FU resistance [89].

Something of interest is that UV-radiation-induced DNA damage stimulates SHMT1 translation. In response to UV-radiation-induced damage, a ribosome is recruited to an internal ribosome entry site (IRES) located in the 5′-untranslated region (UTR) on SHMT1, and SHMT also experiences an increase in SUMOylation after UV treatment, localizing them to the nucleus [84,92]. It was shown that SHMT1 is involved in DNA repair and genome stability after UV exposure. This process makes sense when one considers that thymine is sensitive to UV radiation, likely forming cyclobutane pyrimidine dimers that require nucleotide excision repair or DNA lesion bypass. As mentioned earlier, insufficient pools of thymine are likely to lead to increased uracil incorporation into DNA, leading to genome instability [92]. There has been evidence of SHMT activity relating to radiotherapy resistance in heck neck squamous cell carcinoma [93].

#### Guanosine monophosphate synthase (GMPS)

**Figure 3:**
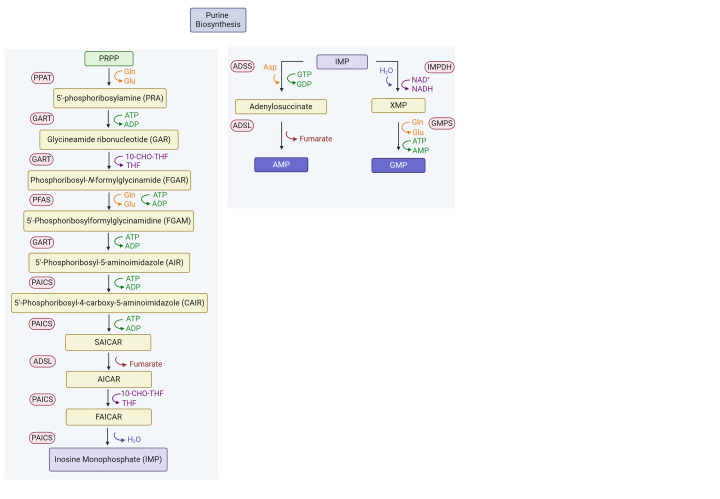
The overall scheme of de novo purine biosynthesis Note that IMP is shown twice due to the importance of its role in the downstream of the pathway.

**Figure 4:**
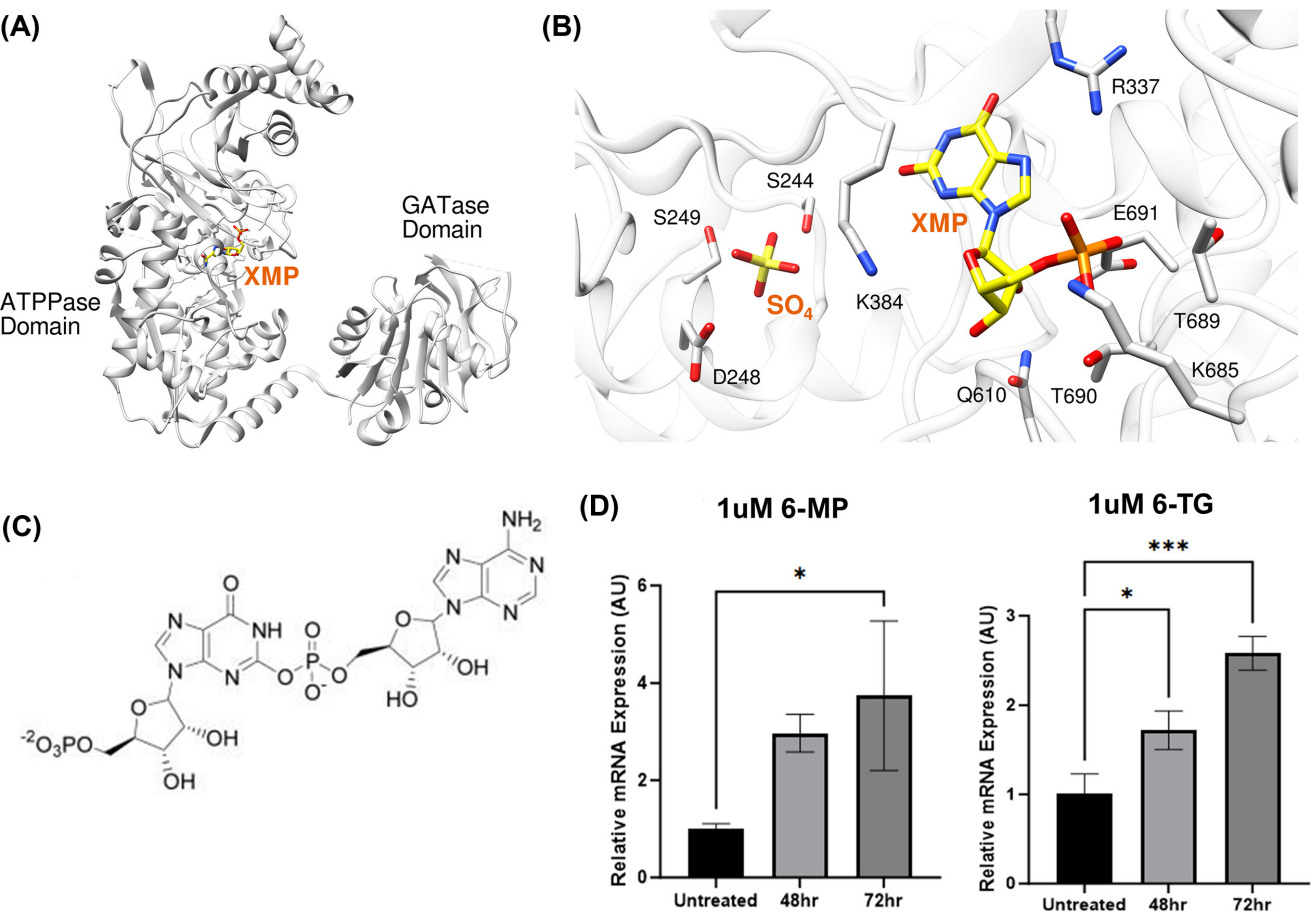
Guanine monophosphate synthase (GMPS) (**A**) The overview of the crystal structure of GMPS (PDB ID: 2VXO). There are two distinct domains in human GMPS, ATPPase domain and GATase domain. (**B**) The substrate of GMPS, XMP, was found to bind in the active site of GMPS along with the sulfate binding pocket where the reaction intermediate, adenylyl XMP, might bind. The figures were generated via Chimera. (**C**) The chemical structure of the key intermediate of GMP biosynthesis via GMPS, adenylyl XMP. (**D**) RT-qPCR data with MOLT-4 cells showed the up-regulation of GMPS upon the treatment of 6-MP and 6-TG (unpublished data from Jung lab).

The purine *de novo* pathways are sequentially performed by six different enzymes, which are co-localized to form the purinosome, to catalyze the conversion of phosphoribosylpyrophosphate (PRPP) to inosine 5′-monophosphate (IMP) (Figure 3) [94]. Purine biosynthesis pathways have been thought to be good cancer drug targets, for there is an excessive need for purine nucleotides including ATP and GTP in the fast-growing cells such as cancer cells. *De novo* purine biosynthesis is reported to be utilized higher in leukemia cell including acute lymphoblastic leukemia (ALL) and acute myeloma leukemia (AML) cells compared with normal bone marrow cells or peripheral blood lymphocytes [95,96]. Guanosine monophosphate synthase (GMPS), which converts xanthosine monophosphate (XMP) into GMP, has two separate domains, and the human GMPS crystal structure (PDB ID: 2VXO) indicates that ATP pyrophosphatase (ATPPase) domain has clearly defined active site (Figure 4A). The substrate XMP was bound in the active site of the ATPPase domain (Figure 4B), and GMPS adenylates XMP by forming a covalent O2-adenyl-XMP intermediate (adenylyl XMP, Figure 4C) [97–99]. GMPS gene is reported to be located on chromosome 3q24 [100], and it was found that GMPS/ubiquitin-specific protease 7 binds to ecdysone-regulated loci and mutation on GMPS exhibited severe mis-regulation of ecdysone target genes [101,102].

GMPS was reported to be up-regulated in various malignant tumors, such as lung squamous cell carcinoma, ovarian serous cystadenocarcinoma and head and neck squamous cell carcinoma [103–105]. Our experiment on the MOLT-4 pediatric ALL cell line showed that GMPS was up-regulated upon the treatment of 6-mercaptopurine (6-MP) and 6-thioguanine (6-TG) (unpublished data, Figure 4D), which suggests that cancer cells might try to boost the purine biosynthesis and replication in response to 6-thiopurine that can be incorporated into DNA and RNA. It was displayed that GMPS participated in radiotherapy resistance and played an important role in inducing cell apoptosis of nasopharyngeal carcinoma [104], and it was also shown that the expression of GMPS significantly affects the invasive capacity of melanoma cells and eventually tumor growth, and angustmycin A, a nucleoside-analog inhibitor of GMPS produced by *Streptomyces hygroscopicus* efficiently suppresses melanoma cell invasion *in vitro* and tumorigenicity in immunocompromised mice [106]. So far, there has not been much research on GMPS as a drug target, and future research will give us more insight into what role this crucial enzyme at the last step of GMP synthesis plays in cancer.

#### Inosine triphosphatase (ITPase)

**Figure 5:**
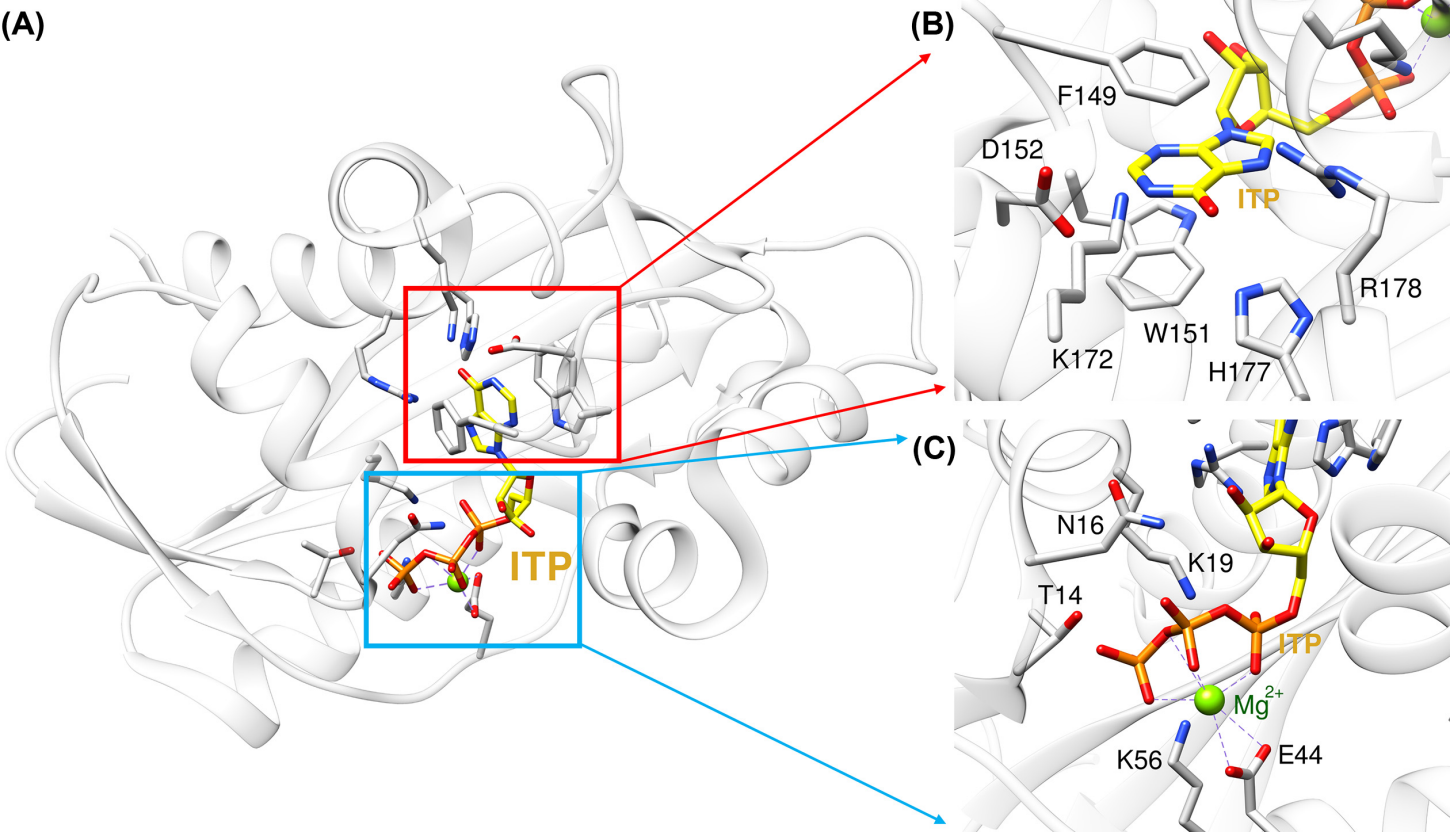
Inosine triphosphatase (ITPase) (**A**) The overall structure of ITPase complexed with ITP (PDB ID: 2J4E). Two separate binding sites are labeled in red and blue. (**B**) The key residues of ITPase interacting with HX in the active site of ITPase are shown. (**C**) The key residues interacting with phosphates in the active site of ITPase are shown.

Inosine triphosphate pyrophosphatase (ITPase) is encoded by the *ITPA* gene. In humans, ITPase is a 45 kDa α/β homo-dimeric protein, and the structure and function of ITPase is highly conserved across all three domains of life [107]. The crystal structure of human ITPase (PDB ID: 2J4E) showed that ITP binds snugly in the active site of ITPase (Figure 5A), and both the hypoxanthine ring (Figure 5B) and triphosphate group (Figure 5C) have hydrogen bonding interactions with the residues of ITPase in the active site.

A total of 11 *ITPA* variants have been reported, two of which have been a focus due to a link to 6-thiopurine intolerance [108]. The main function of ITPase is to hydrolyze inosine triphosphate (ITP), xanthine triphosphate (XTP), and their deoxynucleotide forms (dITP and dXTP) to their monophosphate forms and pyrophosphate. Decreasing the amount of noncanonical purine (d)NTPs can be important in preventing their misincorporation into DNA and RNA. Additionally, the monophosphate forms are important precursors for adenine and guanine in the *de novo* purine synthesis pathway [107,109]. Deoxyinosine triphosphate (dITP) can be incorporated into DNA by various enzymes [110–114], and we recently showed that one of the translesion synthesis (TLS) enzymes, DNA polymerase eta (polη) was able to incorporate dITP into DNA across dC or dT [114]. ITPase has high specificity for ITP/dITP but xanthosine triphosphate (XTP/dXTP) is another substrate of ITPase [115] along with some non-canonical nucleotides including 6-thioguanine (6-TG) tri-phosphate [116]. Genetic defects in the *ITPA* gene are closely related to human diseases including early infantile encephalopathy [117], infantile dilated cardiomyopathy [118], and neural depolarization and epilepsy [119]. The accumulation of ITP/dITP and the imbalance of the nucleotide pool are linked to human diseases including immunodeficiency, Lech-Nyman syndrome, and cancer [120–123], and the level of inosine and other precursors in nucleotide biosynthesis are tightly regulated in cells [122,124,125]. Since the level of non-canonical nucleotides is tightly regulated, disrupting their levels by modulating ITPase can be a novel strategy to combat many diseases including cancer.

Inosine misincorporation in place of guanine RNA is well documented in RNA polymerase II. The incorporation of inosine and guanine across from cytosine has a similar *K*_m_ and *V*_max_, but this mistake is often corrected through proofreading. Inosine misincorporation in mRNA can lead to ribosome stalling and a reduced rate of translation [107,126]. DNA polymerases can also mis-incorporate inosine, albeit much less often than RNA pol II, and recently we showed that DNA polymerase eta (polη) can incorporate inosine via deoxyinosine triphosphate (dITP) into DNA [114]. Xanthine can also be incorporated opposite C, but also at low efficiency. In DNA, inosine can be non-mutagenic since cytosine is usually recruited opposite of inosine. Although, attempted repair of inosine can lead to recombination and chromosomal rearrangements. On the other hand, xanthine can lead to DNA polymerase stalling and is difficult to bypass, likely leading to mutagenesis. Apart from DNA effects, these two nucleotides can negatively affect proteins that utilize ATP and GTP [126].

Production of (d)ITP and (d)XTP can result from the phosphorylation of their monophosphate and diphosphate forms by purine NMP kinases and nucleoside diphosphate kinases. Elevated IMP levels, possibly due to disruption of purine synthesis, can lead to increased IMP phosphorylation and (d)ITP generation. They may also be a result of oxidative and nitrosative deamination of (d)ATP and (d)GTP [107], and ITPase can be a key for dealing with damage that results from oxidative stress. Deamination is one of the major pathways for DNA lesion formation and introduction, and adenine can be deaminated into hypoxanthine (HX) and its various phosphate forms either by external stimulants or spontaneously [127].

*ITPA* has been of interest because mutations resulting in partial loss of ITPase have led to the accumulation of ITP in erythrocytes. Its function requires Mg^2+^ and is favored under reducing alkaline conditions [107,109]. Mutations resulting in the complete loss of ITPase have been reported to cause a fatal infantile neurodevelopmental disorder. Inosine is not detected in DNA, which indicates that the diseases are caused by errors in RNA or inhibition of nucleotide synthesis enzymes [107,128]. ITPA deficiency can cause dilated cardiomyopathy. Variants of ITPA due to aberrant splicing have been known to cause epileptic encephalopathy and other neurological disorders [129]. One study showed that ITPA expression is increased in people exposed to metal-rich particulates, and metal particulates are known to be carcinogenic and are associated with increased formation of reactive oxygen species (ROS) and oxidative stress. DNA is sensitive to ROS, and guanine is particularly sensitive to being oxidized. This indicates that ITPase plays a role in supplying dGTP in the case of guanine damage and repairing oxidative damage of purines [130]. ITPA deficiency can lead to infertility, likely due to accumulated oxidative damage to oocytes, spermatozoa, and embryos [131].

### Base excision repair – uracil DNA glycosylase (UDG/UNG) and methyl-CpG binding domain protein 4 (MBD4)

The human body produces an average of 330 billion cells daily at a rate of 3.8 million per second. This means over 300 billion new DNA is synthesized daily and with this extremely high synthesis rate, enormous errors of over 20,000 lesions/cell/day happen [132]. The body has successfully devised means to counteract these damages and restore the DNA and genome integrity. This is where base excision repair (BER) comes into action. BER is a crucial pathway in humans that restores the chemical integrity of DNA. It’s a way an organism checks, identifies, and corrects damaged DNA. These damages may be caused by endogenous moieties such as DNA polymerase infidelity, ROS, or exogenous agents like UV radiation, chemical mutagens, and plant toxins. These damages often occur as chemical modifications to the DNA, usually in the form of deamination, alkylation, and oxidation. A common example of this modification is the hydrolytic deamination of 5-methylcytosine (5-meC) and cytosine to thymine and Uracil which poses severe problems to the integrity of the genome if not corrected. This spontaneous event occurs at a rate of 200-300 lesions per day, ultimately resulting in the transcription of wrong base pairs in the next round of DNA replication (C: G, T: A) [133].

BER, one of the cell’s mechanisms to tackle DNA damage plays its role of solely removing non-bulky DNA adducts and eliciting its action in humans, Base Excision Repair occurs in two phases, the initial damage-specific stage and the damage-general phase and these phases require the action of a minimum of four enzymes; a damage-specific monofunctional DNA glycosylase that recognizes and excises a damaged base, resulting in the formation of abasic site, a key intermediate for the next enzyme apurinic/apyrimidinic (AP)-endonuclease. AP-endonuclease (APE1) is a multifunctional enzyme that repairs distorted DNA lesions and converts the abasic site into a single nucleotide gap by cleaving the 5′ phosphodiester backbone bond of the DNA to produce 3′ hydroxyl (OH) and a 5′ deoxyribose phosphate groups. These precursors are used in the next phase of BER where DNA polymerase β performs repair synthesis by inserting the appropriate nucleotide after which DNA ligase restores the phosphodiester bond. In this review, we will be focusing on two of the enzymes involved in the damage-specific stage where DNA glycosylase primarily functions- Uracil-DNA glycosylase (UDG) and Methyl-CpG-binding domain (MBD4). BER typically takes place in the nucleus, however, sometimes it may occur in the mitochondria.

Triphosphatases are a diverse group of enzymes or proteins that catalyze the hydrolysis of triphosphate molecules such as ATP to produce monophosphates (AMP in this instance) and release energy or specifically perform cellular functions such as signal transduction and nucleic acid synthesis. Triphosphatases are important in nucleotide biosynthesis in numerous ways. First, in DNA replication DNA polymerases use deoxyribonucleoside triphosphates (dNTPs) to synthesize a new DNA strand complementary to the template strand. Triphosphatases can help regulate the pool of dNTPs available for replication by hydrolyzing excess dNTPs to dNDPs. Second, during transcription in RNA synthesis, ribonucleoside triphosphates (NTPs) are used to create the complementary RNA strand. Finally, Triphosphatases play a role in RNA capping, which is the modification of the 5′ end of mRNA. During this process, a guanosine triphosphate (GTP) is added to the 5′ end of the nascent mRNA molecule and triphosphatases ensure the removal of the γ-phosphate from the GTP molecule during this process, generating a 5′-end cap structure (m7GpppN) that is essential for mRNA stability, translation, and processing [134]. Cells that initiate BER (damage-specific stage) are collectively called glycosylases and there are approximately 11 recognized DNA glycosylases with overlapping functions depending on the type of lesions (U:A) and this type of glycosylase subsequently determines the mechanisms of the damage-general phase. Glycosylases contain amino/carboxyl-terminal extensions that are usually disordered and are required for target DNA recognition [135].

Uracil DNA glycosylase, the first DNA glycosylase enzyme is one of the 4 minimum enzymes utilized during a base excision repair (BER) [136]. It’s been well studied structurally and biochemically and as a result, served as a prototype for other DNA glycosylases. More evidence shows that UDG is important because it has been conserved to humans from bacteria and some viruses and is expressed in almost all cells in humans and its major function is to protect cells from cytotoxicity and mutagenicity [137]. UDG is a family of monofunctional glycosylases encoded by the UNG gene that primarily functions by cleaving mismatched Uracil attached to the DNA which may have occurred due to chemical modifications explained earlier [134]. This single-domain enzyme is hypothesized to flip uracil nucleotides out of the DNA base stack using a ‘Push-Pull’ mechanism whereby the leucine side chain infiltrates the DNA (push mechanism) eliciting complementary interactions from the Uracil binding pocket resulting in the productive binding. UDG recognizes damaged or mismatched base pairs in double-stranded DNA (sometimes even some single-stranded DNA (ssDNA)) and then removes this base lesion suggesting that when uracil bases were present in cells, especially at replication forks [138]. DNA damage uses ATR/chk1cyclin to stop damaged cells in the S or M phase before initiating the repair by UDG [139], and there is a significant accumulation of uracil in the cell, triggering apoptosis in these cells in the absence of UDG. This shows the level of importance of UDG in BER, DNA integrity, and ultimately, cell survival. The disruption of UDG (in knockout mice) is indicated in disease conditions like HIGM syndrome and lymphoid hyperplasia [140].

Methyl-CpG binding domain protein 4 (MBD4) is another major lesion-specific glycosylase we will review in this paper. During DNA damage, some of the bases are exposed to hydrolysis which results in the deamination of 5-methylcytosine (5-meC) and cytosine to thymine and uracil respectively. This modification severely damages the DNA in the severity of mutations and ultimately, diseases. It is then the responsibility of MBD4, a unique multidomain protein, with two DNA domains, an N-terminal methyl binding domain, and a C-terminal glycosylase domain [133]. MBD4 elicits its action by catalyzing the cleavage and removal of T and U paired with Guanine (G) within the CpG site [141]. It binds strongly to heavily methylated foci in the DNA allowing for a base-flipping mechanism to occur where the damaged DNA is yanked out of the DNA helix through a torsional rotation of the phosphodiester backbone into the active site cleft. Zhu et al. showed that MBD4, in conjunction with cleaving 5-meC, also excises 5-hydroxymethyl uracil (5-hmU) from hemi-methylated DNA [141]. *In vitro* studies have shown that loss of the *mbd4* gene does not result in tumorigenesis; however, it results in the formation of a pool of C and T at CpG sites which may result in the predisposition to cancer. In summary, triphosphatases are somewhat linked to BER because the byproduct of their actions (monophosphates like dUMP) is mis-incorporated in the DNA to which enzymes like UDG and MDG4 have flip and kick out of the DNA helix to maintain the integrity of the DNA and prevent disease generation.

### Translesion synthesis – DNA polymerase eta (polη)

Translesion synthesis (TLS) is one of the DNA damage tolerance mechanisms, and a group of specialized DNA polymerases called Y-family DNA polymerases usually perform TLS [142,143]. In humans, polymerase kappa (polκ), iota (polι), eta (polη), and Rev1 are the reported members of Y-family DNA polymerases, and TLS requires other components such as proliferating cell nuclear antigen (PCNA) and polζ, which is consisted of Rev3, Rev7, polθ, and polν. There also is a known prokaryotic Y-family polymerase, Dpo4 [144,145], and all the known Y-family DNA polymerases, both prokaryotic and eukaryotic, share common catalytic domains, which are finger, thumb, palm and little finger domains [146].

This section will focus on one of the Y-family DNA polymerases, polymerase eta (polη) and its potential role in drug resistance toward nucleotide analog drugs such as 5-FU. Polη (UniProt ID: Q9Y253) was first recognized from the group of patients of Xeroderma Pigmentosum (XP) variant syndrome, who showed hypersensitivity toward many skin diseases including skin cancer, and this abnormal hypersensitivity was closely related to the defect in DNA repair for thymidine dimer caused by UV [147–150]. Later, it was revealed that XP variant syndrome patients all carry some mutations in the *POLH* gene, which encodes polη [151]. Though polη is an error-prone DNA polymerase without exonuclease activity, which is capable of proof-reading [152], polη was reported to be able to bypass TT cyclobutene pyrimidine dimer (CPD) with no significant error [153]. However, the bypass by polη across many other lesions such as 8-oxoguanine is mostly still error-prone, and the biochemical and structural insights into the error-prone lesion bypass by polη are well compiled in a couple of reviews [146,154]. The first crystal structure of the catalytic domain of polη, complexed with the CPD containing DNA and the incoming nucleotide, was published in 2010 [155]. Ever since, there have been a lot of publications presenting polη crystal structures in complex with various DNA lesions such as oxoG [156], oxoA [157,158], abasic site [159], cisplatin adduct [160], N7-alkylguanine [161–163], and hypoxanthine/xanthine [113], and there have been several reports regarding the factors that contribute to the pro-mutagenic bypass of polη such as *syn-anti* conformational equilibrium, catalytic metals, and enol-keto tautomerism [114,154].

**Figure 6:**
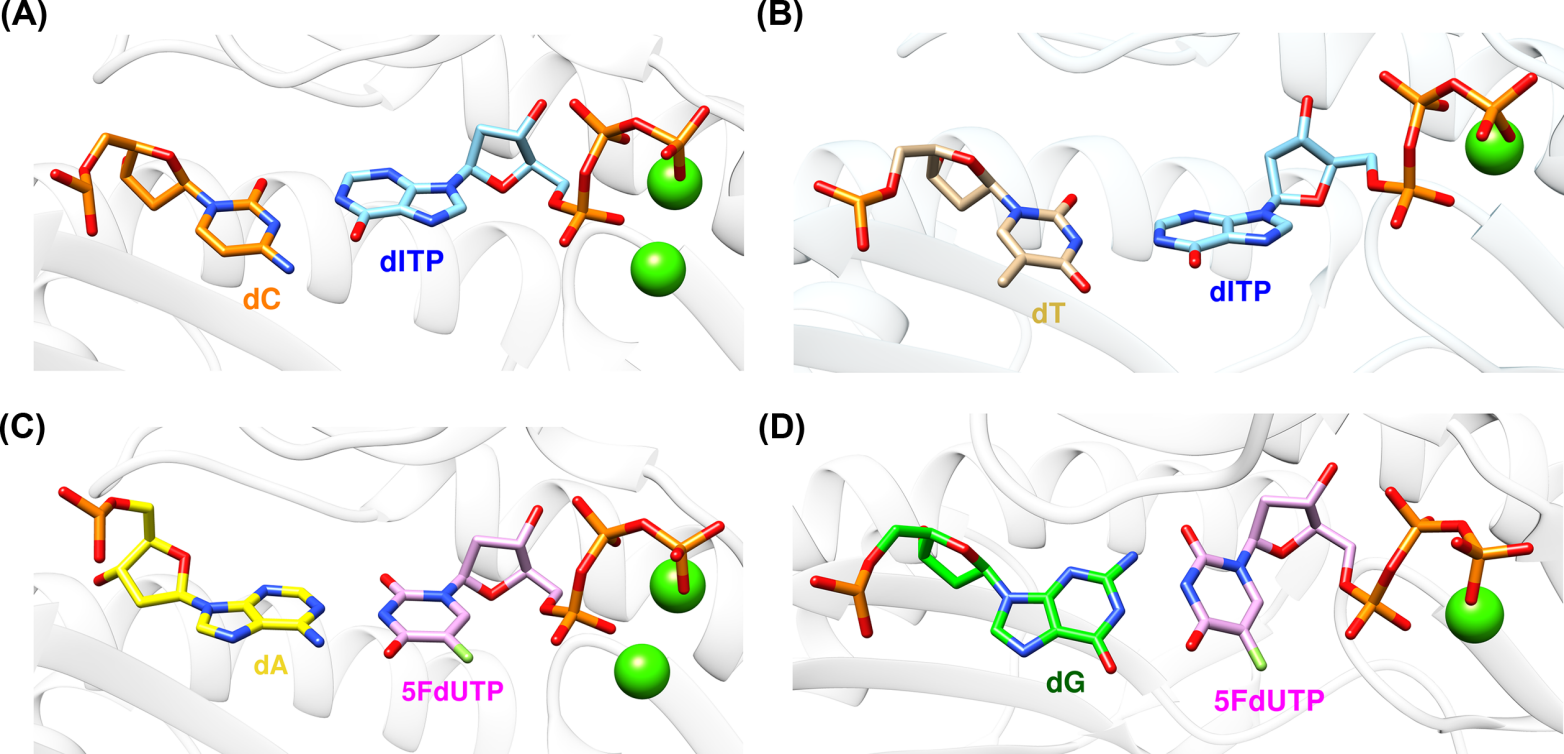
Base pairing interactions involving DNA lesions in the active site of polη (**A**) The crystal structure of polη complexed with dITP opposite dC (PDB ID: 8FN3) shows Watson-Crick base pair between dC and dITP. (**B**) The crystal structure of polη complexed with dITP opposite dT (PDB ID: 8FOG) shows a non-optimal Watson-Crick like base pair between dT and dITP (**C**) The crystal structure of polη complexed with 5FdUTP opposite dA (PDB ID: 8GKR) shows Watson-Crick base pair between dA and 5FdUTP. (**D**) The crystal structure of polη complexed with 5FdUTP opposite dG (PDB ID: 8GML) shows distorted Watson-Crick like base pair between dG and 5FdUTP.

Recently, it was also shown that polη is capable of incorporating deoxyinosine triphosphate (dITP) across dA and dG in a promutagenic fashion [114]. One of the common DNA lesions is deamination, and the nitric oxide-induced or spontaneous deamination of cytosine (C), adenine (A) and guanine (G) can form uracil (U), hypoxanthine (HX) and xanthine (XT), respectively [164,165]. HX and XT were efficiently bypassed by some of the DNA polymerases including translesion synthesis (TLS) human DNA polymerase eta (polη) with high mutagenicity [113]. HX, which is a base for inosine, is known to act similarly with guanine in DNA base pairing [166], and it forms a favorable Watson-Crick base pair with cytosine almost exclusively over unfavorable Hoogsteen base pair with thymine, which causes A to G mutation as a result [167,168]. For example, the ratio between dCTP incorporation over dTTP opposite HX was reported to be 55:1 to 120:1 in various DNA polymerases [113,169]. The mutagenic potential of HX was also displayed in human cell lines such as HEK293 and HCT116 cell lines as well [170], and the lagging strand was more susceptible to mutation than the leading strand in HEK293 cell lines. The crystal structures of polη-dC:dITP showed that the cytidine ring and HX ring have Watson-Crick base pair in the active site of polη (Figure 6A), while polη-dT:dITP showed that thymidine ring and HX ring have a distorted Watson-Crick like base pair in the active site of polη (Figure 6B). Nucleotide insertion assay also confirmed that polη incorporated dITP across dC and dT with 27:1 ratio (7.17 for dC vs. 0.52 for dT in terms of *k*_cat_*/K*_m_), which is much more mutagenic compared with replicative DNA polymerases. These results revealed that the incorporation of dITP into DNA can be done by polη and that this incorporation of inosine by polη can be promutagenic (via incorporation across dT). Combined with the previously reported results of the bypass of HX by polη [113], it is clear that polη is closely related to the incorporation and bypass of inosine in DNA.

Not just inosine, we also reported that 5-FU can be incorporated into DNA by polη [171]. In the earlier section, we discussed the role of 5-FU and its metabolism, and the fact that it can be directly incorporated by one of the TLS polymerases implies that DNA lesion bypass might be closely related to the drug metabolism and resistance of nucleotide analog drugs including 5-FU. In our recent report, the crystal structures of polη-dA:5FdUTP showed that adenine ring in the template and 5-FU ring in the incoming 5FdUTP have Watson-Crick base pair in the active site of polη (Figure 6C), while polη-dG:5FdUTP showed that thymidine ring and guanine ring have a distorted Watson-Crick like base pair in the active site of polη (Figure 6D). Nucleotide insertion assay also confirmed that polη incorporated 5FdUTP across dA with similar efficiency with dTTP incorporation (10.3 for 5FdUTP:dA vs. 13.6 for dTTP:dA in terms of *k*_cat_*/K*_m_). Polη was shown to incorporate 5FdUTP across dG with higher efficiency than dTTP incorporation (0.25 for 5FdUTP:dG vs. 0.16 for dTTP:dG in terms of *k*_ca__t_*/K*_m_). It indicated that polη incorporated 5FdUTP across dA and dG with 40:1 ratio (10.3 for dA vs. 0.25 for dG in terms of *k*_cat_*/K*_m_), which is mutagenic compared with the incorporation of dTTP. These results, along with the incorporation of dITP by polη, revealed that the incorporation of 5FdUTP, and potentially other nucleotide analogs, into DNA can be done by polη and that this incorporation of a non-canonical nucleotide into DNA by polη can be promutagenic. These results also suggest that polη and TLS are closely related to the drug action, metabolism, and resistance of the nucleotide analog anticancer agents, and TLS and polη can be utilized to combat drug resistance in cancer, which depends heavily on nucleotide biosynthesis for its replication and survival.

## Conclusion

In summary, we have discussed several enzymes in nucleotide biosynthesis, DNA repair, and DNA lesion bypass that have been relatively under-investigated in this review. Fast-growing cells have huge demand for replication and thus for nucleotides, and those cells including cancer cells inevitably make higher errors during the replication, which results in bringing in DNA repair and bypass processes to ensure the completion of replication to prevent the possibility of apoptosis. The enzymes and pathways we covered here are highly relevant for overcoming the prevalent drug resistance problem in nucleotide analog anticancer agents and for developing novel drugs. More research needs be done to fill the current knowledge gap between nucleotide biosynthesis and DNA lesion repair/bypass especially in cancer cells, yet the enzymes and pathways discussed in this review could be new keys to overcoming drug resistance and developing novel anticancer drugs.

## Abbreviations

AP: apurinic/apyrimidinic
APE1: AP-endonuclease
BER: base excision repair
CPD: cyclobutene pyrimidine dimer
CRC: colorectal cancer
GTP: guanosine triphosphate
MBD4: methyl-CpG-binding domain
PCNA: proliferating cell nuclear antigen
PRPP: phosphoribosylpyrophosphate
UDG: Uracil-DNA glycosylase
XP: Xeroderma Pigmentosum
